# Two years survival of primary cardiac leiomyosarcoma managed by surgical and adjuvant therapy

**DOI:** 10.1186/s13569-017-0069-3

**Published:** 2017-03-09

**Authors:** K. Behi, M. Ayadi, E. Mezni, K. Meddeb, A. Mokrani, Y. Yahyaoui, F. Ksontini, H. Rais, N. Chrait, A. Mezlini

**Affiliations:** Medical Oncology Department, Salah Azaeiz Institute, Tunis, Tunisia

**Keywords:** Leiomyosarcoma, Cardiac tumors, Survival, Chemotherapy

## Abstract

**Background:**

Cardiac tumors are a very rare entity. Leiomyosarcoma represents less than 1% of cases.

**Case presentation:**

a 51-year-old woman diagnosed with primary left atrium leiomyosarcoma. She was treated by optimal surgery and adjuvant chemotherapy. She is still alive after a follow-up of 24 months without evidence of local or distant recurrence.

**Conclusions:**

Cardiac leiomyosarcoma is a rare tumor with a dismal prognosis. Surgery is the mainstay of treatment. Adjuvant treatment is still controversial.

## Background

**Fig. 1:**
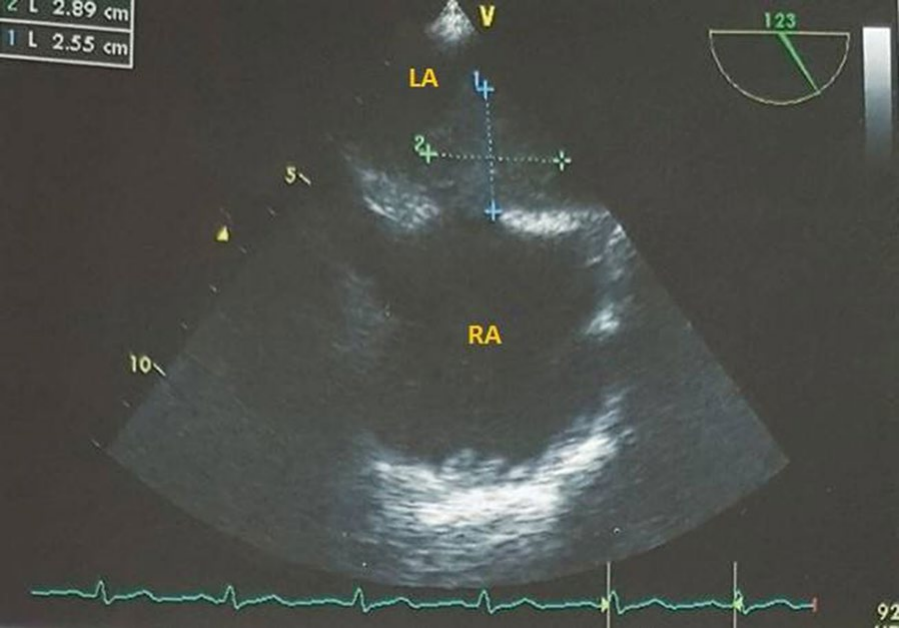
Transoesophageal echocardiography showing mid esophageal 20° view. Left atrium tumor. *RA* right atrium, *LA* left atrium

Cardiac tumors are a very rare entity with an incidence of 0.02%. Only 25% are malignant with a prevalence ranging from 0.001% to 0.28 [1]. Primary cardiac cancers are scarcer than cardiac metastases [2]. Most frequent histologic types are Angiosarcomas followed by rhabdomyosarcomas, mesotheliomas and fibrosarcomas. Leiomyosarcoma represents less than 1% of cases [3]. In spite of improvement of multidisciplinary treatment including surgery, chemotherapy and radiotherapy, the prognosis remains (Fig. 1) poor with a median survival of 6 months [3].

This case aimed to describe the clinical, histological, therapeutic and prognostic features of this rare pathology.

## Case report

**Fig. 2:**
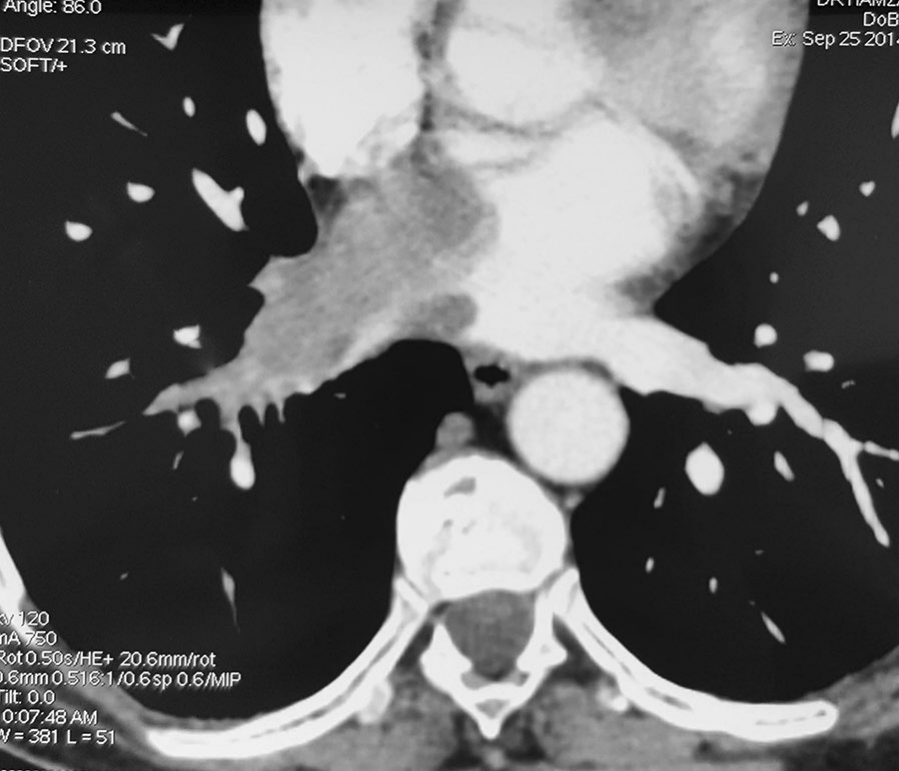
Computed tomography; Transverse section: 6 cm defect involving the right atrium and the right inferior pulmonary vein, which appears markedly enlarged

We present the case of a 51-year-old woman, with past medical history of hypertension, who consulted in September 2014 for bilateral lower limb pain and lower extremity edema since 6 months. The diagnosis of hypertrophic osteoarthropathy was suspected. In this context, a CT scan revealed a 6 cm defect, involving the right atrium and the right inferior pulmonary vein, which appears markedly enlarged. This aspect evoked a large intracavitary thrombus. A transesophageal cardiac ultrasound showed a 30 × 26 mm, with little mobility, lobed tumor, in the left atrium, connected to the atrial septum. The tumor wasn’t obstructive. Cardiac chambers dimensions and pulmonary pressure were normal. There was no systolic or diastolic left ventricular dysfunction. The patient was referred to a cardiovascular surgeon with a suspected diagnosis of left atrium myxoma. Surgery was performed through median sternotomy. The patient had a cardiopulmonary bypass (CPB) with aortic and bi-caval cannulation. The left atrium was dissected, revealing a voluminous, septal based tumor involving the right inferior pulmonary vein and the posterior wall of the left atrium, suggestive of malignancy. We did a wide en bloc excision of the tumor, extended from the posterior wall of the left atrium Fig. 2 and inferior pulmonary vein to pericardial reflection. Reconstruction of the left atrium with two pericardial patches (an anterior septal and posterior parietal) was achieved according to Sutureless de lacourt-Gayettechnique. Macroscopic examination showed a friable whitish mass measuring 40 × 30 × 30 mm.

Microscopic examination revealed the presence of conjunctival tumoral proliferation made of spindle cells with a fascicular organization infiltrating myocardial fibers. A high mitotic activity was noticed. Surgical margins were clear. Immunohistochemical staining showed an intense and diffuse positivity of alpha-smooth-muscle actin and caldesmon and the negativity of PS100, desmine and myogenin. Based on these findings, diagnosis of primary cardiac leiomyosarcoma grade 3 according to FNNCLCC was confirmed.

Post-operative CT scan revealed no metastases. According to a multidisciplinary staff, an adjuvant chemotherapy consisting on six cycles of Doxorubicin and Ifosfamide was prescribed.

The patient is regularly followed. After a follow-up of 24 months, she still has no clinical or radiological evidence of recurrence.

## Discussion

We presented a rare case of cardiac leiomyosarcoma treated by surgery followed by an adjuvant chemotherapy. The patient is still alive after a follow-up of 24 months.

Until December 2015, we found only 32 cases of primary cardiac leiomyosarcoma with available data. In most studies, it was defined as tumors originating only from cardiac chambers, excluding those located in the pericardium and great vessels [4]. Epidemiological, clinical, therapeutic features and outcomes were listed in the Table 1.

**Table 1:** Epidemiological, clinical, therapeutic features and outcomes of reported cases of primary cardiac leiomyosarcomas

	Author	Age	Sex	Symptom	Site	Size (cm)	Surgery	Chemotherapy	Radiotherapy	Survival (months)
1	Kornberg [8]	21	F	N	RA	6	R2	Doxorubicin–ifosfamamide	N	3
2	Takamizawa [9]	53	M	Cough Inferior limbs oedema	RA	3	N	N	N	0.5
3	Fox [10]	61	M	Chest pain Vomiting	LV	9	R2	N	N	6
4	Ishitoya [11]	26	F	Dyspnea Inferior limbs oedema	LA	7	R0	N	N	5
5	Hattori [11]	19	M	Dyspnea Hemoptysis	LA	13	R0	N	Y	2
6	Minakata [12]	69	F	Dyspnea Cyanosis	LA	6	R0	N	N	3
7	Pins [13]	29	F	Chest pain	RV	3	R0	N	N	NA
8	Pins [13]	25	M	Syncope	RA	9	R0	Doxorubicin–Ifosfamide—Actino-Doxorubicin	Y	60
9	Minardi [14]	67	M	Dyspnea Cough	LA	8 and 2	R0	N	N	7
10	Burnett [15]	60	F	Dyspnea	RV	NA	R2	N	N	0
11	Andersen [16]	86	F	Pulmonary oedema	LA	4	R0	N	N	15
12	Ogimoto [17]	73	F	Dyspnea	LA	NA	R0	N	N	3
13	Willaret [18]	70	F	Dyspnea Chest pain Palpitation	RV	4	R0	N	N	7
14	Strina [19]	33	F	Dyspnea Syncope	LA	8	R0	Doxorubicin–Ifosfamide	Y	29
15	Lee [20]	45	F	Dyspnea	RV	8	R2	Doxorubicin–Ifosfamide	N	NA
16	Malyshev [21]	43	F	Pulmonary oedema	LA	NA	R2	Doxorubicin–Ifosfamide	N	15
17	Smith [22]	40	F	Dyspnea	LA	7	R1	N	Y	18
18	Antunes [23]	53	F	Pulmonary oedema	LA	NA	R2	Metoxantrone Dacarbazine Cyclophosphamide	N	6
19	Rastan [24]	76	F	Dyspnea	RV	6	R1	N	N	NA
20	Antunes [23]	33	F	Anorexia Tirednes, Dyspnea Chest tightness	RA	4	R0	N	N	5
21	Astarcıoğlu [25]	40	F	Dyspnea	RV	9	R0	Doxorubicin	N	42
22	Glaoui [26]	47	M	Pulmonary oedema Dyspnea	RA	5	R0	Doxorubicin–Ifosfamide	N	7
23	Nakanishi [27]	74	M	Dyspnea	LA	NA	R0	N	Y	8
24	Wilbring [28]	43	M	Paravertebral back pain Dyspnea	LA	6	R0	N	Y	9
25	Parissis [29]	36	M	Dyspnea	RA	NA	R0	Doxorubicin–Ifosfamide	N	NA
26	Mazzolla [30]	21	F	N	LA	7	R0	Doxorubicin–Ifosfamide- cisplatin	N	24
27	Esaki [31]	68	M	Dyspnea	RV	NA	R0	N	N	2
28	Guschmann [32]	61	F	Dyspnea	LA	NA	R0	Y	N	NA
29	Davis [33]	65	F	Dyspnea Chest pain	LA	6	R0	Y	N	18
30	Panday [34]	67	M	Dyspnea	RV	NA	R0	N	N	36
31	Pessotto [35]	24	M	Syncope Atrial fibrillation	LA	7	R1	Doxorubicin–Ifosfamide—Actino-Doxorubicin	Y	84
32	Lo [36]	28	F	Long term fever Body weight loss	LA	5	R0	Y	N	5

*RA* right atrium, *LA* left atrium, *RV* right ventricle, *LV* left ventricle, *R0* macroscopically complete resection, *R1* resection with microscopic margins, *R2* resection with macroscopic margins, *N* no, *Y*, yes, *NA*, not available

Median age at diagnosis was 48 with a female predominance [4].

This tumor has a poor prognosis due essentially to advanced stages at presentation. It usually remains asymptomatic until advanced stages. Even when it becomes symptomatic, presentation is atypical and non specific. Symptoms of obstruction especially dyspnea is found in 78.1% of cases [4]. Physical examination isn’t helpful no more. In our case, the patient was asymptomatic and the diagnosis was suspected fortuitously on the findings of a CT performed for another aim.

Echocardiography, especially the trans-esophageal route, is habitually the initial imaging modality. It may show the tumor, its extent and its hemodynamic consequences (Fig. 1). CT scan and cardiac MRI provide further information about morphology, location and extent of the mass (Fig. 2). Cardiac MRI is more efficient to evaluate myocardial involvement. CT scan is useful to assess extracardiac extent and metastasis [5].

Cardiac leiomyosarcomas have a high rate of local and distant recurrence, occurring even after an optimal resection of the primary tumor.

The left atrium is the most frequent location of cardiac leiomyosarcomas (51%). That joins operative and radiological findings, in our case.

Biopsy is the gold standard for histological confirmation but this step can sometimes be overtaken and the diagnosis is then made on the examination of resected mass.

Complete surgical resection, when it’s possible, is the mainstay of treatment.

Since soft tissue sarcoma is a heterogeneous group, benefits of adjuvant chemotherapy isn’t clearly established. Several studies defined subgroups associated with a high risk of local and distant relapse. Risk varies depending on factors like size >5 cm, high grade, depth and chemosensitive histologies with a metastatic potential. Leiomyosarcoma belongs to the high risk group. Major drugs used are Doxorubicin, Ifosfamide and Dacarbazine [6]. It’s what leaded us to propose six cycles of Doxorubicin and Ifosfamide in adjuvant setting to our patient, after a multidisciplinary team consultation.

Indications of radiation therapy are mostly restrained to palliative setting. It’s proposed when margins of resection are positive or for aggressive localized disease or for recurrences. Since the lack of evidence concerning the efficacy of radiotherapy in management of cardiac leiomyosarcomas and the poor tolerance, its use stills equivocal and unusual.

According to the reported cases of leiomyosarcomas with cardiac involvement, the mean survival time of patients who underwent surgery and chemotherapy was about 12 months [6]. Several factors are suspected to enworse the prognosis particularly the high grade, a high mitotic index, positive surgical margins and metastasis [7].

In the case above, our patient had a grade 3 tumor with a high mitotic activity. She underwent a carcinologic surgery and adjuvant chemotherapy. Currently, she is still alive and there is no evidence of local recurrence or metastasis, after a follow-up of 24 months.

## Conclusions

Cardiac leiomyosarcoma is a rare tumor with a dismal prognosis. Wide margin resection is the mainstay of treatment. For soft tissue sarcoma, adjuvant chemotherapy is still controversial. Nevertheless, leiomyosarcoma belongs to the high risk group of soft tissue sarcoma associated with a metastatic potential. In this group, there is a trend to outcomes improvement with adjuvant chemotherapy. Drugs used in this context are Doxorubicin, Ifosfamide and Dacarbazine.

Regarding the scarcity of this disease, it’s important to report all cases with a longer follow-up to refine indications and modalities of adjuvant treatment and prognostic factors.
